# Bone age is not just for kids

**DOI:** 10.7554/eLife.66916

**Published:** 2021-03-02

**Authors:** Jane A Cauley, Dolores M Shoback

**Affiliations:** 1 Department of Epidemiology, Graduate School of Public Health, University of PittsburghPittsburghUnited States; 2 Endocrine Research Unit, San Francisco Department of Veterans Affairs Medical Center, Department of Medicine, University of California, San FranciscoSan FranciscoUnited States

**Keywords:** osteoporosis, fragility fracture, post-fracture mortality, multistate model, osteoporotic fracture, Human

## Abstract

More informed discussions between physicians and older adults about the consequences of an initial osteoporotic fracture could encourage more patients to consider treatments that protect against future fracture.

**Related research article** Ho-Le TP, Tran TS, Bliuc D, Pham HM, Frost SA, Center JR, Eisman JA, Nguyen TV. 2021. Epidemiological transition to mortality and re-fracture following an initial fracture. *eLife* **10**:e61142. doi: 10.7554/eLife.61142

Chronological age and "bone age" are the same in most people, but sometimes they are different. For decades pediatricians have used bone age – which can be estimated from X-rays – as a tool to assess health and development in children (Creo and Schwenk, 2017). For physicians treating the elderly, improved methods for estimating bone age of older adults would be helpful when assessing the risk of osteoporotic fractures: this is important because osteoporosis is under-diagnosed, under-treated and under-appreciated as a factor that influences both life expectancy and quality of life. Now, in eLife, Thao Phuong Ho-Le, Tuan Nguyen and colleagues at the Garvan Institute of Medical Research in Sydney and other institutions in Australia and Viet Nam report that they have developed a model that can estimate bone age in older adults and provide improved estimates of the risks of subsequent osteoporotic fractures and death following an initial fracture (Ho-Le et al., 2021).

The data come from a well-established population-based study, the Dubbo Osteoporosis Epidemiology Study, which has been following around 3500 men and women in Dubbo, a city in south-west Australia, who were 60 or over in 1989. Ho-Le et al. developed a multi-state model to provide prediction estimates for fracture, refracture and death. In this model individuals can be in one of five states – no fracture, first fracture, second fracture, third fracture and death – and can transition through all five states, or move directly from any of the first four states to death. Ho-Le et al. report that, during the 20 year follow-up, the risk of a second fracture was higher in women (36%) than in men (22%), but the mortality risk was higher in men (41%) than women (25%). The risk of transitioning from any state to death was also much higher in men than women.

As mentioned above, chronological age and bone age are usually the same. But given a low bone mineral density coupled with other risk factors for fracture, the age of your bones can be greater than your chronological age. Physicians use a tool called the Fracture Risk Assessment tool (FRAX) to decide if a patient should receive treatment to protect against osteoporotic fractures: in general, if the probability of hip fracture over the next ten years is 3% or higher, or if the risk of a major osteoporotic fracture (that is, a fracture to the spine, forearm, hip or shoulder) is 20% or higher, treatment is recommended. While the 3% risk threshold for hip fracture prevention was deemed cost-effective when FRAX was developed (Tosteson et al., 2008), a patient might think: "But I have a 97% chance of not fracturing". However, if the physician could reply, "You may be 70, but you have the bones of an 80 year old", the patient may be more willing to consider treatment.

The results of this study are important for other reasons. Existing risk assessment tools do not take into account the increased chances of further fractures, let alone death (Rubin et al., 2013), but the model developed by Ho-Le et al. can estimate the 5 year individual probability of transitioning from no fracture to fracture or to death. For example, for a 70-year-old woman with low bone mineral density but no other risk factors, the probability of transitioning from no fracture to first fracture (10%) was similar to the risk of death (8.6%). However, once she experiences a first fracture, the risk of another fracture goes up dramatically (16.5%) and exceeds the risk of dying (10.4%). With this information, the patient may be more likely to consider treatment.

There are several unanswered questions and inherent limitations. Older folks fear institutionalization, so the possibility of transitioning to disability outcomes and assisted living could be added to the model. The Dubbo study is also a single cohort from one city, so the model needs to be validated in cohorts around the world. Moreover, since Dubbo residents are 98% white, the model needs to be tested in other race/ethnicities. Lastly, the model is only adjusted for comorbidities at baseline. It is highly likely that the participants developed other chronic diseases over the 20 year follow-up, but such diseases are not included in the model, so the risk of refracture and death may have been underestimated.

Screening for high-risk patients who may benefit from therapy is important because prevention of future fractures and their consequences is possible with the armamentarium of treatments that are available. Future pragmatic randomized clinical trials are needed to test whether screening in the community, using this type of multistate model, can increase treatment rates and ultimately reduce fractures and their consequences.
